# Cognitive behavioral therapy for insomnia helps to reverse cognitive impairment in insomnia patients

**DOI:** 10.5935/1984-0063.20210026

**Published:** 2022

**Authors:** Daniela Deyanira Guarneros Roniger, Yoaly Arana Lechuga, Enrique Esqueda León, Rosa Obdulia González Robles, Óscar Sánchez Escandón, Guadalupe Jovanna Terán Pérez, Javier Velázquez Moctezuma

**Affiliations:** 1 Universidad Nacional Autónoma de México, Programa de Maestría y Doctorado en Psicología - Coyoacan - Mexico.; 2 Universidad Autónoma Metropolitana, Sleep Disorders Clinic - Iztapalapa - Mexico.; 3 Universidad Autónoma Metropolitana, Mathematics Department - Iztapalapa - Mexico.; 4 American British Cowdray Medical Center, Neurological Center - Santa Fé Campus - Mexico.; 5 Universidad Autónoma Metropolitana, Area of Neurosciences, Department of Biology of Reproduction, CBS - Iztapalapa - Mexico.

**Keywords:** Sleep Initiation and Maintenance Disorders, Cognitive Therapy, Cognition, Memory, Attention, Executive Function

## Abstract

**Introduction:**

Insomnia is the most common of sleep disorders, it induces a wide variety of organic symptoms, including somatic and cognitive impairments. There are pharmacological drugs nowadays that help diminish sleep impairments due to insomnia. However, most of them seem to be worsening cognitive impairments, benzodiazepine receptor agonists, in particular, seem to induce an even worst deterioration of cognitive function. On the other hand, cognitive behavioral therapy for insomnia (CBT-I) has shown to be a reliable tool to improve the whole picture of insomnia.

**Objectives:**

To analyze the effect of CBT-I on insomnia symptoms and cognitive performance in patients suffering from chronic insomnia.

**Material and Methods:**

Ten subjects with a diagnosis of insomnia and no pharmacological treatment were evaluated pre- and post-six biweekly sessions of CBT-I with two neuropsychological batteries, BANFE and NEUROPSI attention and memory.

**Results:**

CBT-I significantly improves both the symptoms of insomnia, measured subjectively with a sleep diary and the Athens insomnia scale, and the cognitive performance measured with the neuropsychological batteries.

**Discussion:**

CBT-I is not only an effective tool for the treatment of insomnia but also helps to ameliorate cognitive performance.

## INTRODUCTION

Insomnia is one of the most common sleep disorders. It is defined as a difficulty or persistent complaint in initiating and/or maintaining sleep or waking up too early. As consequence, patients experience daytime symptoms that are directly related to nighttime sleep difficulty. Chronic insomnia refers to the presence of these symptoms for at least three months, three times per week^1^. It has been reported that insomnia may lead to a decrease in quality of life. Between 30 to 48% of the general population, may present at least one insomnia symptom throughout their life, with approximately 6% of them diagnosed with an insomnia disorder^2,3^. In Mexico, the PLATINO study^4^ reports an insomnia prevalence of 35% in adults over 40 years old, being more frequent in women (41.8%) than in men (25.7%). The risk factors for insomnia are age, female gender, medical or psychiatric disorders, shift work, being unemployed, or low socioeconomic status^2,5,6^.

Also, people who suffer from insomnia, often complain about daytime impairments of cognitive performance. There is evidence of impairment in memory, attention, and some executive functions due to insomnia symptoms^7,8^. Nevertheless, treatments for insomnia currently available are not oriented to improve cognitive symptoms.

Pharmacological handling of insomnia includes benzodiazepine receptor (BZR) agonists as the first-line treatment. BZR is widely prescribed nowadays; however, recent reports indicate that both acute and chronic administration induced additional cognitive impairments in psychomotor function, attention, working memory, episodic memory, and metacognition^9^. It has been suggested that those effects are mediated by α1GABAA and α5GABAA receptor mechanisms, and that α1GABAA receptor mechanism appears to be sufficient for impairments in executive function induced by BZR; meaning that not only classical BZR like clonazepam, but also more selective BZR like Zolpidem or Zaleplom can cause cognitive impairments^10^, and these effects could last for more than six months after withdrawal^11^.

On the other hand, non-pharmacological treatments for insomnia, including transcraneal magnetic stimulation (TMS) and cognitive behavioral therapy for insomnia (CBT-I), have been shown to result in an effective amelioration of insomnia clinical picture^12,13^. However, TMS is not yet an easily available procedure. Instead, CBT-I is widely used and has proved to be an efficient treatment for insomnia, with long-term benefits on sleep. CBT-I includes different kind of techniques that improve sleep parameters, such as sleep latency (SOL), number of awakenings (NWAK), wake after sleep onset (WASO), time in bed (TIB), total sleep time (TST), and sleep efficiency (SE)^12-15^.

CBT-I is a psychological therapeutic approach that makes mention of a set of behavior and thought modification techniques designed to improve sleep. Among these techniques, those used in the intervention of this study were: basic sleep education, sleep restriction therapy, sleep hygiene, stimulus control therapy, and cognition therapy (for more details consult: behavioral treatments for sleep disorders). However, nowadays there is no solid evidence about recoveries of cognitive impairments once the CBT-I has improved sleep parameters. Therefore, this study aimed to assess the effects of CBT-I on some cognitive functions after modifying some sleep parameters.

## MATERIAL AND METHODS

### Subjects

Participants were selected from insomnia patients of the Sleep Clinic of the Universidad Autónoma Metropolitana (UAM). Thirty-eight adults (age 23-54 years) with a diagnosis of chronic insomnia according to the ICSD-3, and no current pharmacological treatment were initially included in the study. Sleep disorders specialists confirmed the insomnia diagnosis and ruled out the presence of any other sleep, medical, psychiatric and neurological disorder. The inclusion criteria were insomnia diagnosis, no current clinical treatment, not taking any drug that had shown to cause cognitive deficits at the moment of the evaluation and for at least the minimum withdrawal period of each drug (none of the participants took any drug for at least 2 years before the study), no evidence of medical, psychiatric or neurological disorders, and no major depression symptoms according to the Beck depression inventory. Additionally, climacteric women were not included in the study. Participants signed the informed consent form. The institutional ethics committee for research approved the protocol of the study with the number 1901.

### Measures

**Sleep parameters:** insomnia and somnolence were evaluated with the Athens insomnia scale (AIS) and the Epworth sleepiness scale (ESS), respectively^16,17^. Therefore, participants were asked to complete sleep diaries at home every morning until 2 weeks after ending the CBT-I. The sleep diary offers the following parameters: SOL, NWAK, WASO, TIB, TST, and SE, as well as a subjective rating of sleep quality (SQ) on a ten-point Likert scale (1 = poorest sleep quality and 10 = best sleep quality).

**Polysomnography (PSG):** PSG recording was conducted on a single night on Cadwell equipment. To detect electroencephalographic (EEG) abnormalities, EEG was recorded with all the electrodes from the 10-20 system, using a bipolar and longitudinal montage. All the other recordings were conducted according to the American Academy of Sleep Medicine. Participants arrived at 8:30 p.m., went to bed from 10:00 p.m. until 6:00 a.m. Recordings were scored by qualified technicians according to standard criteria. The parameters obtained included measures of sleep continuity (i.e., SOL, WASO, NWAK, TST, TIB, and SE), as well as frequency and duration of sleep stages.

**Cognitive performance:** cognitive functions were evaluated with two neuropsychological batteries that had been designed and validated for the Mexican population. NEUROPSI attention and memory, and neuropsychological battery of frontal lobes and executive functions (BANFE). These batteries evaluate attention, working memory, episodic memory, executive functioning, and metacognition with different tasks.

### Procedure

The selected volunteers were asked to answer the AIS, ESS, and BDI. Then the PSG was performed to objectively confirm the absence of any neurological abnormalities or another sleep disorder. The morning following the PSG record, participants were evaluated for cognitive performance with the two batteries described earlier; these batteries were counterbalanced for each participant, between each battery there was a break of 20 to 30 minutes. At that moment, participants were instructed to fulfill the sleep diary every morning for 10 weeks, and CBT-I started after they fulfilled the first two weeks of sleep diary.

Once the patients were evaluated and have fulfilled two weeks of SD, CBT-I was started. The therapy was delivered individually, in 6 biweekly sessions, each lasting 45 to 60 minutes. The techniques used were basic sleep education, sleep restriction therapy, sleep hygiene, stimulus control therapy, and cognitive therapy. The sessions were organized as follows:

Session 1: basic sleep education; sleep restriction therapy according to Spielman et al. (1987)^18^; four individualized rules of sleep hygiene; stimulus control therapy, and cognition therapy, finding the dysfunctional cognitions of the patient, and asking them to keep an outstanding and concerns diary.

Session 2, 3, 4, and 5: assessment of the biweekly work, reinforcement of the weakest changes the patient had made, clarification of every doubt or difficulty regarding adherence, the addition of new sleep hygiene rules, modification of the previous sleep schedule, as suggested by Spielman et al. (1987)^18^, and cognitive therapy confronting dysfunctional sleep cognitions with previous information and with the sleep diary, and exchanging them with more rationale substitutes (one or two thoughts per session).

Session 6: closure of the therapy, showing the patient all the benefits he or she had obtained in quantity and quality of sleep, and reinforcing the main techniques that will help avoid relapse in insomniac behaviors.

Two weeks after the last CBT-I session, participants were evaluated for cognitive performance with the same batteries as pre-treatment, and insomnia and somnolence symptoms with the AIS and EES, respectively.

Statistical analysis was done using a t-test comparing all the sleep and the cognitive parameters measured before and after CBT-I, establishing *p* significance at <0.05.

## RESULTS

From the initial sample of 38 subjects, only 20 fulfilled the inclusion criteria, and ten of them completed the study. Four males and six females. Age 24 to 52 years, with a scholar average of 16.1 years.

No EEG abnormalities were found in any of the participants included. Despite the insomnia complaints, the PSG parameters showed a mild sleep impairment, since the average of total sleep time was 395.97 min (±37.34) (6.59 hrs.), sleep efficiency 81.14 % (±8.03), sleep latency 21.6 min (±7.37), and WASO 63 min (±41.53).

Regarding subjective sleep parameters that were measure with sleep diary, we found moderate to severe impairment at baseline, as well as significant improvements of the following parameters: sleep efficiency increased 13.86% from 75.83 to 89.69%, sleep latency decreased 36.23 minutes (from 51.9 pretreatments to 15.67 posttreatment), number of awakenings decreased 0.94 per night, and the overall sleep quality increased from 6.83 to 7.93. We also found a 25.5-minute increase in total sleep time and a 15.53-minute reduction in total wake time; however, those changes were not statistically significant. There was a significant reduction of 50.74 minutes in bed, and this is a common effect of CBT-I given that excessive time in bed results in insomnia symptoms (t statistic and *p*-value of each analysis is reported in Table 1A).

**Table 1:** The table shows the values on sleep parameters before and after CBT-I.

A.-				
**Sleep Diary**	**Baseline X (S.D.)**	**Post-treatment X (S.D.)**	**T**	**p (unilateral)**
Time in bed (min)	509.8 (46.98)	459. 07 (57.92)	-2.714	0.012
Total, sleep time (min)	386.8 (69.20)	412.25 (59.16)	1.006	N.S.
Sleep efficiency (%)	75.83 (14.46)	89.69 (5.31)	2.877	0.009
Sleep latency (min)	51.9 (31.62)	15.67 (5.48)	-3.719	0.002
# Awakenings	1.55 (1.15)	0.61 (0.50)	-3.338	0.004
Total, wake time (min)	23.57 (27.75)	8.04 (8.52)	-1.674	N.S.
Overall sleep quality	6.83 (1.35)	7.93 (1.13)	3.780	0.002
**B.-**				
**Questionaires**	**Baseline (X ± S.D)**	**Post-treatment (X ± S.D)**	**T**	**p (unilateral)**
Athens insomnia scale	18.1 (5.28)	6.7 (3.129)	-5.326	0.001
Epworth sleepiness scale	3.9 (4.33)	2.5 (3.27	-2.143	0.030

The Athens sleep scale showed a significant reduction in global score, which means that patients have fewer complaints about their sleep-in terms of time and quality, as well as daytime impact. There was also a significant decrease in global scores of the Epworth sleepiness scale, even though sleepiness is not necessarily a symptom of insomnia (Table 1B).

**Figure 1:**
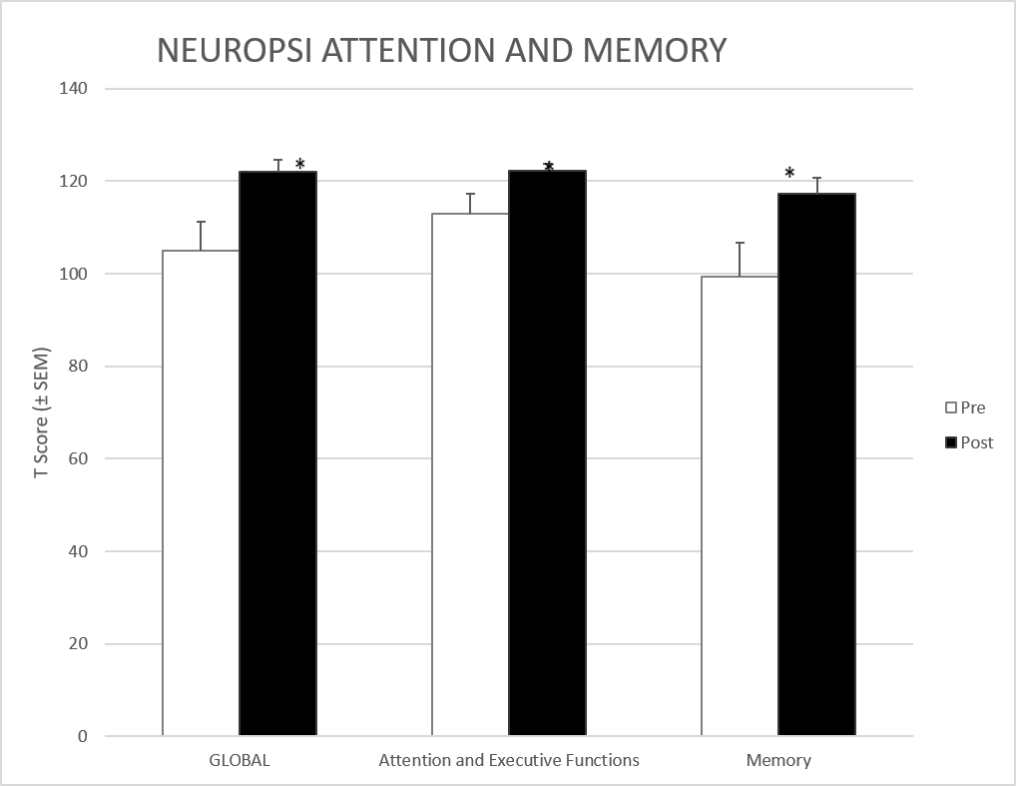
Scores on NEUROPSI attention and memory pre and post treatment.

Concerning cognitive changes after CBT-I, results showed a significant improvement in almost all the subscales of the tests applied. Figure 1 shows results concerning the changes on the NEUROPSI attention and memory. As can be observed, CBT-I induced significant improvements in global score, attention, executive functions, and memory. It is worth mentioning that at baseline, the average global score, as well as the subscales scores were within the class of normal cognitive function (85-115), and after the CBT-I, they changed to the rank of high normal cognitive function (>116).

Table 2 shows the results in those cognitive parameters evaluated with NEUROPSI attention and memory that were statistically significant or were close to. We found an improvement in phonological verbal fluency, as well as a decline in time participants take to respond Stroop test; both tasks are part of the attention and executive functions subscale. There was also an improvement in different memory tasks, both in coding, and evocation.

**Table 2:** The table shows the results in those cognitive parameters evaluated with NEUROPSI attention and memory that were statistically significant or were close to.

Neuropsi attention and memory	Baseline X (S.D.)	Post-treatment X (S.D.)	T	p (unilateral)
Global	105 (19.641)	122.100 (8.225)	4.551	0.001
Attention And	113 (13.31)	122.200 (4.80)	2.194	0.028
Executive				
Functions				
Phonological	3.5 (0.707)	3.000 (0.816)	-3.000	0.007
verbal fluency				
Stroop (time)	3.8 (0.421)	4.1 (.316)	1.964	0.040
Memory	99.3 (23.414)	117.200 (11.252)	2.703	0.005
Verbal memory (spontaneous)	8.7 (1.828)	9.6 (2.170)	2.211	0.027
Verbal memory (keys)	8.6 (1.955)	9.9 (2.024)	2.176	0.028
Rey-Osterrieth (evocation)	20.4 (6.095)	26.2 (3.155)	4.468	0.001
Themes (coding)	4.5 (0.849)	4.9 (0.316)	1.809	0.051 NS
Names (evocation)	6.3 (2.162)	7.2 (1.475)	2.0769	0.033
Story	10 (2.538)	11.4 (1.505)	1.8005	0.052 NS

**Figure 2:**
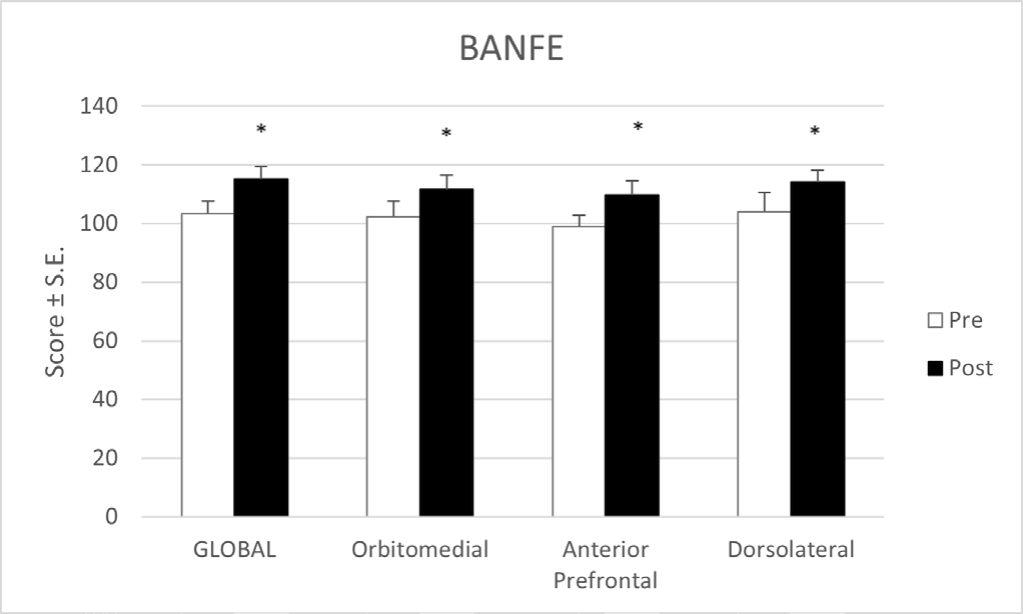
Scores on BANFE pre and post treatment.

Regarding the test of executive functions and frontal lobe (BANFE), results showed also significant improvements both in the global score as in all its subscales (Figure 2). In this case, the baseline scores and the post-treatment scores were both in the normal cognitive function rank; however, they were a statistically significant improvement.

Table 3 shows the results obtained in each of the items and subscales of the BANFE test that were statistically significant or were close to. We found an improvement in the tasks that measure risk behavior (cards game: risk percentage and cards game score), semantic classification (number of total categories and abstract categories, an average of animals per category, and total score), and cognitive flexibility (cards classification: hits and time).

**Table 3:** The table shows the results obtained in each of the items and subscales of the BANFE test that were statistically significant or were close to.

Banfe	Baseline X (S.D.)	Post-treatment X (S.D.)	T	p (unilateral)
Global	103.4 (13.517)	115.2 (13.414)	3.827	0.002
Orbitomedial	102.3 (16.640)	111.7 (15.188)	1.948	0.041
Risk percentage	3.8 (1.398)	4.9 (0.316)	2.538	0.015
Cards game (score)	3.4 (1.646)	4.8 (0.421)	2.688	0.012
Anterior prefrontal	98.9 (12.187)	109.8 (14.875)	2.739	0.001
Semantic classification (abstract categories)	3.2 (1.032)	4 (1.154)	2.228	0.026
Metamemory (negative 4 (1.247) errors)	4 (1.247)	4.8 (0.421)	1.809	0.051 NS
Dorsolateral	104 (11.460)	114.1 (12.922)	3.377	0.004
Cards classification (hits)	47.5 (10.211)	54.6 (3.475)	2.551	0.015
Cards classification (time)	4 (0.942)	4.9 (0.316)	3.250	0.004
Semantic classification (number of categories)	3.8 (0.918)	4.8 (0.632)	3.354	0.004
Semantic classification (total score) total	3.4 (0.966)	4.2 (1.032)	2.228	0.026
Semantic classification (average of animals)	4.7 (0.483)	4 (0.666)	-3.279	0.004

Regarding the test of executive functions and frontal lobe (BANFE), results showed also significant improvements both in the global score as in all its subscales (Figure 2).

## DISCUSSION

The results obtained support the notion that CBT-I is a reliable tool to ameliorate complaints of insomnia. Concerning cognitive impairments of insomnia, results also indicate that CBT-I significantly improves some cognitive parameters in insomnia participants of this study.

Despite initial complaints of insomnia, PSG recording supports the diagnostic of mild insomnia, mainly because sleep efficiency was less than 85%. It must be mentioned that the PSG study is not the common practice to corroborate the clinical impression of insomnia^1^. Some authors do not indicate a PSG for diagnosis of insomnia. Several reasons support this notion, among others the so-called first night effect. Due to not yet elucidated reasons, patients sleep differently in the lab than in their homes, and very often, insomniac patients sleep better in the lab. Thus, it is common that patients who complain of insomnia show normal sleep parameters^19-22^. Nevertheless, complaints of subjective deficiencies in sleep are the common feature in insomniac patients. Furthermore, these sleep impairments often have a significant correlation with impairments of cognitive functioning^9,20^.

In the present sample, complaints of insomnia are present along with cognitive impairment. Also, CBT-I was capable of modifying the subjective perception of sleep. Patients reported a significant improvement in the perception of the quality of their sleep after CBT-I and this change is parallel with a significant improvement of cognitive functioning.

In the subjects examined, all the subscales, as well as the global scale explored by the NEUROPSI attention and memory and BANFE batteries showed a significant improvement. This means that CBT-I improved the performance of executive functions linked to the frontal lobe.

It has been previously described that insomnia is associated with decreased regional cerebral blood flow (rCBF) to the frontal medial, occipital, and parietal cortices, and the basal ganglia and that behavioral therapy is associated with a reversal in the cerebral deactivation of some of this brain regions, including frontal cortex^23^. It has also been described a decrease in brain metabolism in the prefrontal cortex of insomniac patients during wake^24^, as well as a reduced volume in the orbitofrontal cortex^25^, dorsolateral prefrontal cortex^26^, and hippocampus^27^; those structures play a major role in attention, memory and executive functions, and the anatomical and functional changes could be the underlying reason for the complaints on cognitive impairment in patients with insomnia. Therefore, the reversibility in those structural and anatomical changes by the CBT-I^23,24^ could be the reason for the improvement in cognitive functions as memory, attention, and executive functions, that are shown in this study.

Even though insomnia is frequently associated with cognitive deficits, available pharmacological treatments do not have a beneficial influence on this deficit but on the contrary, common hypnotic drugs worsen the cognitive deficit. Thus, the present results support the notion that CBT-I is an excellent option for the treatment of insomnia because it modifies the subjective perception of sleep deficits and, according to the present results, also improves the cognitive impairment that is present in insomniac patients.

In conclusion, insomnia is a highly disabling disorder due, in large part, to the cognitive impairment it causes in patients. This deterioration prevents any functional person from achieving their maximum potential in the domains of psychological, social, leisure, vocational, or daily functioning; therefore, it impairs the quality of life. One of the main reasons why people with insomnia seek treatment is precisely because of the deterioration in their quality of life as a consequence of poor functioning in memory, attention, problem-solving, decision-making, and other executive functions. In the present study, it is shown that the treatment for insomnia improves the cognitive functioning of the patients; this without requiring additional treatments that could represent more time and money for the patient. Finally, some components of CBT therapy, mainly the cognitive components, may be useful to control anxiety and mood disorders that are highly frequent in such patients.

Although the present study added some interesting information, regarding the management of insomnia using CBT-I, it must be acknowledged that some limitations should be addressed in future studies. The size of the sample should be increased using the same protocol. Currently, we are permanently increasing the sample size and the results will be disclosed as soon as the results are available.
